# Identification of Key Differentially Expressed Transcription Factors in Glioblastoma

**DOI:** 10.1155/2020/9235101

**Published:** 2020-06-15

**Authors:** Gang Qin, Beiquan Hu, Xianfeng Li, Rongjie Li, Youshi Meng, Yimei Wang, Donghua Zou, Feng Wei

**Affiliations:** ^1^Department of Neurosurgery, The Fifth Affiliated Hospital of Guangxi Medical University, Nanning, Guangxi 530022, China; ^2^Department of Neurology, The Fifth Affiliated Hospital of Guangxi Medical University, Nanning, Guangxi 530022, China

## Abstract

Glioblastoma (GBM) is the most frequent malignant brain tumor in adults. Our study focused on uncovering differentially expressed genes (DEGs) and their methylation in order to identify novel diagnostic biomarkers and potential treatment targets. Using GBM RNA-sequencing data from The Cancer Genome Atlas (TCGA) database, DEGs between GBM samples and paracancer tissue samples were analyzed. Enrichment analysis for DEGs and transcription factors (TFs) was performed. A total of 1029 upregulated genes and 1542 downregulated genes were identified, which were associated mainly with multiple tumor-related and immune-related pathways such as cell cycle, mitogen-activated protein kinase signaling pathway, leukocyte transendothelial migration, and autoimmune thyroid disease. These DEGs were enriched for 174 TFs, and six TFs were differentially expressed and identified as key TFs in GBM: HOXA3, EN1, ZIC1, and FOXD3 were upregulated, while HLF and EGR3 were downregulated. A total of 1978 DEGs were involved in the regulatory networks of the six key differentially expressed TFs. High expression of EN1 was associated with shorter overall survival, while high expression of EGR3 was associated with shorter recurrence-free survival. The six TFs were differentially methylated in GBM samples compared with paracancer tissues. Our study identifies numerous DEGs and their associated pathways as potential contributors to GBM, particularly the TFs EN1, EGR3, HOXA3, ZIC1, FOXD3, and HLF. The differential expression of these TFs may be unlikely driven by aberrant methylation. These TFs may be useful as diagnostic markers and treatment targets in GBM, and EN1 and EGR3 may have predictive prognostic value.

## 1. Introduction

Glioblastoma (GBM) derives from astrocytes and is the most frequent malignant brain tumor, accounting for 60% of brain cancers in adults [1, 2]. The initial therapeutic approach for GBM is surgery, but it is not curative and is applied normally in conjunction with radiotherapy and chemotherapy [3]. However, tumor progression and recurrence occur in many GBM patients, and their survival may be only 12–15 months, with only 10% probability of surviving for 5 years [4].

Similar to other tumors, the tumorigenesis of GBM is driven by genetic [5], epigenetic, and environmental factors [6]. Dysfunction of transcription factors (TFs), which controls the rate of production of messenger RNA (mRNA), can lead to imbalance in homeostasis and thereby to a variety of diseases, including cancer. Some TFs have been shown to be differentially expressed between GBM and normal brain tissue. In fact, a set of four TFs (LHX2, MEOX2, SNAI2, and ZNF22) has been shown to predict the prognosis of patients with GBM effectively [7]. Aberrant methylation of gene promoters appears to contribute to a broad variety of cancers, including GBM. Generally, hypermethylation in CpG islands promotes carcinogenesis, while hypomethylation activates transcription of oncogenes and reduces transcription of tumor suppressor genes [8]. GBM has been associated with hypermethylation of CpG islands in the genes encoding retinoblastoma protein, phosphatase and tensin homolog, and TP53 [9]. Epigenetic silencing of the gene encoding O6-methylguanine-DNA methyltransferase has been associated with longer survival times in GBM patients treated with alkylating agents [10].

Our understanding of the genetic and epigenetic variations that promote GBM initiation and development is poor. Clarifying these variations may contribute to the identification of potential biomarkers for early diagnosis as well as therapeutic targets. As a step in this direction, the present study applied a bioinformatics approach to analyze the DNA methylation profile of GBM using gene data in The Cancer Genome Atlas (TCGA) [11] and Genotype-Tissue Expression (GTEx) databases.

## 2. Materials and Methods

### 2.1. Data Processing

RNA-sequencing data (displayed as read counts) of GBM samples (dataset ID: TCGA-GBM.htseq counts) were downloaded from the TCGA database [12] (https://www.cancer.gov/). The dataset contained 169 GBM tissue samples and 5 paracancer tissue (brain tissue located near cancerous tissue) samples. The mRNAs were extracted according to the *Homo_sapiens.GRCh38.97.chr.gtf* file, downloaded from the Ensembl website (http://asia.ensembl.org/). The current study adhered to the TCGA publication guidelines and data access policies.

### 2.2. Screening of Differentially Expressed Genes (DEGs) and Bidirectional Hierarchical Clustering

DEGs between GBM tissue samples and paracancer tissue samples were analyzed using the *edgeR* package in R software [13]. Fold changes (FCs) in the expression of individual genes were calculated, and genes with |log2FC| > 2 and a *P* value (adjusted by the false discovery rate) <0.001 were considered to be differentially expressed. Bidirectional hierarchical clustering [14] was applied to these DEGs based on Euclidean distance, and the results were displayed as a heat map.

### 2.3. Functional Enrichment Analysis

The *clusterProfiler* package of R software [15] was applied to analyze and visualize functional profiles of genes and gene clusters based on Gene Ontology (GO) [16] and the Kyoto Encyclopedia of Genes and Genomes (KEGG) [17]. A *P* value (adjusted by false discovery rate) <0.05 was considered significant.

### 2.4. TF Target Enrichment Analysis and TF-Target Gene Network

The Database for Annotation, Visualization, and Integrated Discovery (DAVID, https://david.ncifcrf.gov/) UCSC_TFBS annotation tool (v6.8) was used for TF target enrichment analysis [18]. *P* values (adjusted by Benjamini method) <0.05 were considered statistically significant. If the enriched TF was also a DEG, which may play a crucial role in the transcriptional regulation of GBM formation, it was defined as a key differentially expressed TF (KDETF). A TF-target gene network was constructed, and *Cytoscape* software 3.7.1 [19] was used for network visualization.

### 2.5. Validation of Differential Expression Analysis, Survival Analysis, and Differential Methylation Analysis

The Gene Expression Profiling Interactive Analysis (GEPIA) tool (http://gepia.cancer-pku.cn/) [20] was used to combine the brain gene expression profile from the GTEx database [21] and TCGA paracancer tissue data in order to validate the aberrant expression of the KDETFs. The association of overall survival (OS) and recurrence-free survival (RFS) with the key differentially expressed TFs was analyzed by the Kaplan–Meier survival method and compared using the log-rank test in GEPIA. In addition, 34 GBM and 13 normal samples were extracted from another independent dataset (GSE50161) [22] and used to validate the aberrant expression of the KDETFs.

To explore whether the altered expression of key differentially expressed TFs is caused by aberrant DNA methylation, differentially methylated CpG islands of these TFs were screened using the Wanderer tool (http://maplab.imppc.org/wanderer/) [23]. Differences with *P* < 0.05 were considered statistically significant.

## 3. Results

### 3.1. DEGs and Bidirectional Hierarchical Clustering

**Figure 1:**
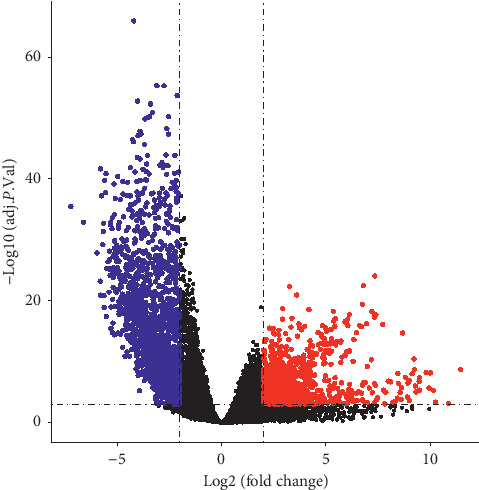
Volcano plot of differentially expressed gene analysis. Red dots represent upregulated genes, blue dots represent downregulated genes, and black dots represent genes not differentially expressed.

**Figure 2:**
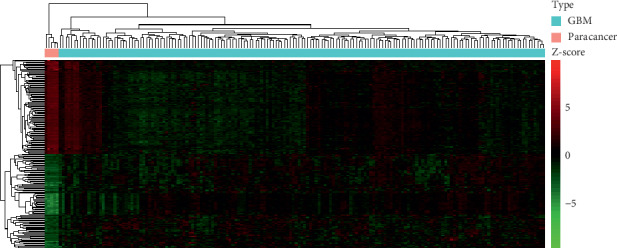
Hierarchical clustering dendrograms of expression patterns of genes differentially expressed between glioblastoma (GBM) and paracancer tissues.

According to the cutoff criteria (*P* < 0.001 and |log2FC| >2), 2571 DEGs were identified between the GBM and paracancer tissue samples, including 1029 upregulated and 1542 downregulated genes (Figure 1). Hierarchical clustering showed that the expression patterns of the 100 most upregulated and 100 most downregulated DEGs (Supplementary Table 1) could accurately distinguish GBM and paracancer tissue samples (Figure 2).

### 3.2. Biological Functions of DEGs

**Figure 3:**
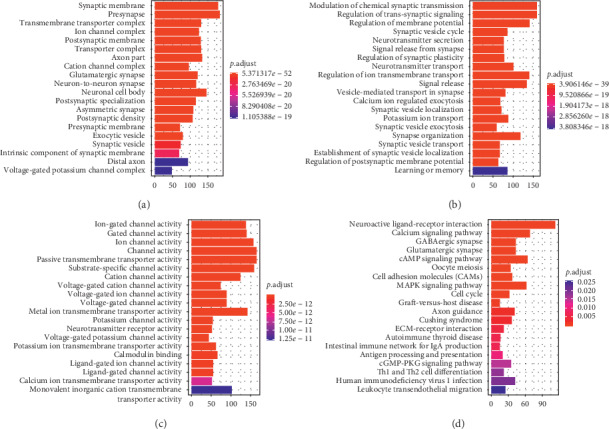
Functional enrichment analysis of differentially expressed genes in glioblastoma in terms of (a) cellular components, (b) biological processes, (c) molecular functions, and (d) Kyoto Encyclopedia of Genes and Genomes Pathways.

To analyze the biological classification of DEGs, GO and KEGG pathway enrichment analyses were performed. GO annotation results were divided into three groups: cellular components (Figure 3(a)), biological processes (Figure 3(b)), and molecular functions (Figure 3(c)). The most significant GO terms (ranked by *P* value) are shown in Figure 3. The DEGs were significantly involved in multiple tumor-related and immune-related pathways (Figure 3(d)), such as cell cycle, mitogen-activated protein kinase (MAPK) signaling, leukocyte transendothelial migration, and autoimmune thyroid disease.

### 3.3. A GBM-Related TF-Target Gene Transcriptional Regulatory Network

**Figure 4:**
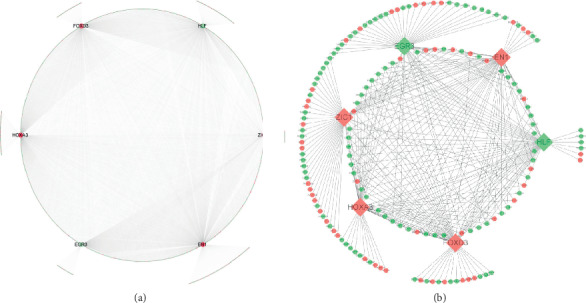
Transcription factor-target gene regulatory networks in glioblastoma. The networks are shown with (a) all their target genes or (b) a subset of 50 target genes randomly selected using the sample function in R. Red color indicates upregulation, while green color indicates downregulation.

A total of 174 TFs were enriched based on the analysis of DEGs using the DAVID UCSC_TFBS annotation tool (Supplementary Table 2). Six of these TFs were themselves differentially expressed in GBM and were, therefore, identified as KDETF: homeobox A3 (HOXA3), engrailed homeobox 1 (EN1), Zic family member 1 (ZIC1), and forkhead box D3 (FOXD3) were upregulated, while hepatic leukemia factor (HLF) and early growth response protein 3 (EGR3) were downregulated. A total of 1978 DEGs were enriched in the regulatory networks of these six key differentially expressed TFs. The aberrant expression of some DEGs may be driven by the key differentially expressed TFs. Thus, we constructed a GBM-related TF-target gene transcriptional regulation network based on these six TFs (Figure 4(a)). For clearer visualization, we also provided a regulatory network for each key differentially expressed TF and a subset of 50 of its target genes randomly selected using the *sample* function in R (Figure 4(b)).

### 3.4. EGR3 and EN1 Expression Associated with Prognosis in GBM

**Figure 5:**
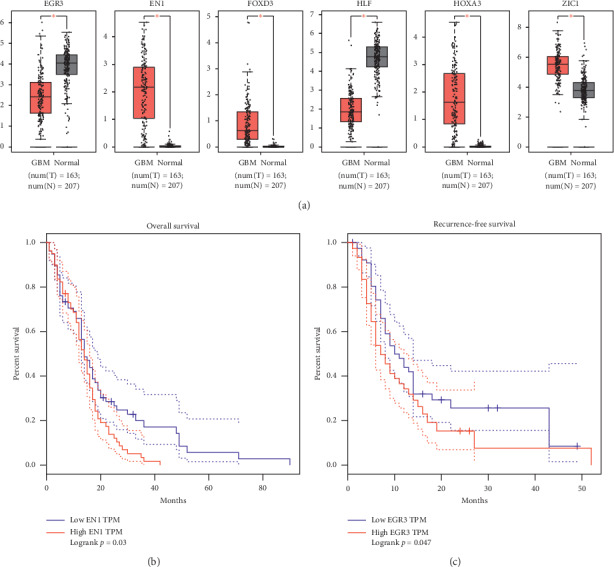
Validation of aberrant expression and survival analysis. (a) The aberrant expression of the six key differentially expressed transcription factors was validated by combining the brain gene expression profile from Genotype-Tissue Expression database and The Cancer Genome Atlas in paracancer tissues. The asterisk indicates *P* < 0.01. (b) High expression of EN1 was associated with shorter overall survival of GBM patients. (c) High expression of EGR3 was associated with shorter recurrence-free survival of GBM patients.

**Figure 6:**
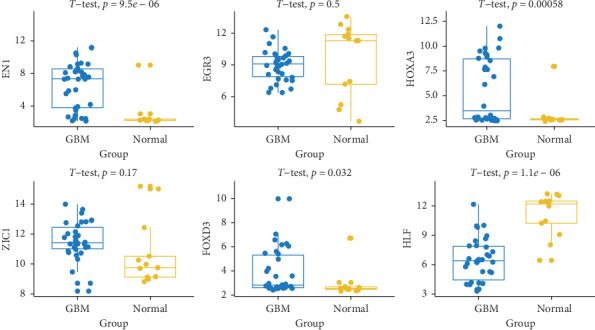
Validation of the aberrant expression of the six key differentially expressed transcription factors in the independent dataset GSE50161.

**Figure 7:**
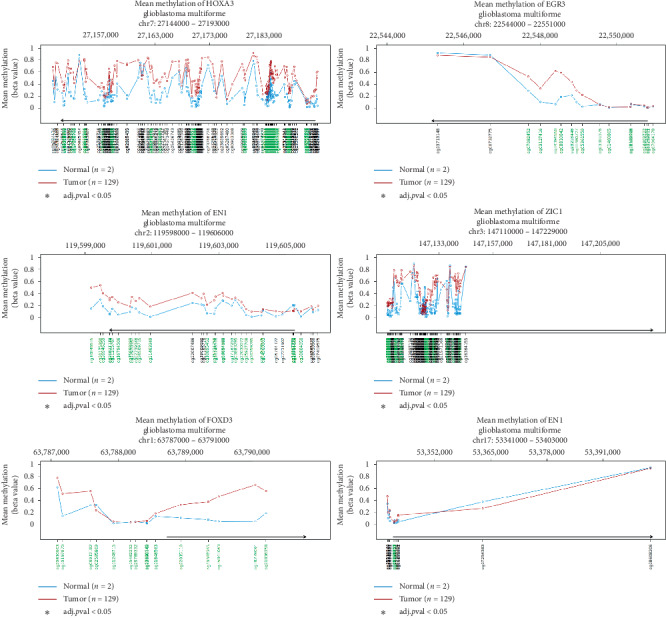
Wanderer analysis of CpG island methylation in the six key differentially expressed transcription factors. Methylation patterns did not differ significantly between GBM and paracancerous tissues.

The aberrant expression of these six key differentially expressed TFs was validated by combining the brain gene expression profile from the GTEx database and data from TCGA paracancer tissue (Figure 5(a)). The correlation between the expression of key differentially expressed TFs and OS or RFS was explored using the GEPIA tool. GBM patients were divided into high and low TF expression groups. High expression of EN1 was associated with significantly shorter OS (Figure 5(b)), while high expression of EGR3 was associated with shorter RFS (Figure 5(c)). The aberrant expression of these six KDETFs was confirmed in the GSE50161 dataset (Figure 6). Analysis with the Wanderer tool showed that methylation of CpG islands in the six KDETFs did not differ significantly between GBM and paracancer tissues (Figure 7), suggesting that differential methylation may not drive aberrant expression of these genes in GBM.

## 4. Discussion

In GBM samples, 1029 genes were upregulated and 1542 genes were downregulated in comparison with paracancer tissues. These DEGs were predominantly involved in multiple tumor-related and immune-related pathways, such as cell cycle, MAPK signaling, leukocyte transendothelial migration, and autoimmune thyroid disease, which may suggest that the occurrence of GBM is associated with an abnormal immune status. In particular, innate immune cells including microglia and macrophages may play a subtype-specific role in GBM by favoring tumor growth and invasion [24]. Besides macrophages, many other immune cells are present in GBM parenchyma, with T cells being, perhaps, the most abundant lymphoid cells. Relatively, few tumor-killing CD8^+^ cytotoxic T cells are present; these cells make up less than a quarter of all CD3^+^ cells [25]. CD3^+^ T cells from GBM patients respond less to direct anti-CD3 stimulation than the corresponding cells from healthy controls *in vitro*, suggesting immunosuppression in GBM [25].

The aberrant expression of genes in GBM and other cancers may be at least partly driven by aberrant expression of TFs because these factors turn genes “on” and “off” to ensure their expression in the appropriate cells at the appropriate times [26, 27]. Our study demonstrated that six TFs were differentially expressed in GBM: HOXA3, EN1, ZIC1, and FOXD3 were upregulated, while HLF and EGR3 were downregulated. A total of 1978 DEGs were enriched in the regulatory networks of these six keys differentially expressed TFs, which may indicate that the aberrant expression of some DEGs may be regulated by these TFs during the development of GBM. Though we failed to validate the aberrant expression of EGR3 and ZIC1 as there are fewer samples in the GSE50161 dataset, the aberrant expressions of EGR3 and ZIC1 are validated in Figure 5(a) which combined brain gene expression profile from TCGA and GTEx database with a large number of samples. Moreover, we constructed a TF-target gene transcriptional regulatory network for GBM. OS and RFS were both explored for each of the five key genes. However, only EGR3 expression was associated with OS, and EN1 expression was associated with RFS. High expression of EN1 was associated with shorter OS, while high expression of EGR3 was associated with shorter RFS, suggesting that these two TFs may be useful as biomarkers of GBM prognosis. Although little is known about the role of EN1 and EGR3 in GBM, previous work showed that EN1 is a hub gene and that its downregulation significantly reduced the viability of triple-negative breast cancer cell lines [28]. Moreover, EN1 may predict risk of brain metastasis in patients with triple-negative breast cancer [29, 30]. EGR3 expression has been shown to be significantly lower in gastric cancer tissues than in matched nontumor tissues, and patients with lower EGR3 expression show poorer prognosis than those with higher expression [31]. EGR3 is downregulated by microRNA 718, and EGR3 expression is significantly reduced in various hepatocellular carcinoma cell lines and tissues [32].

Methylation in CpG islands in the six KDETFs in our study did not differ significantly between GBM and paracancer tissues, which suggest that their differential expression may not be driven by aberrant methylation. This contrasts with several previous studies linking GBM to aberrant methylation. A previous study proposed that DNA demethylation is involved in the recurrence and progression of GBM [33]. Another study reported that 616 CpG sites differentially methylated between glioblastoma and control brain (nonneoplastic brain tissues obtained from patients undergoing surgery for chronic epilepsy), 25% of which were differentially expressed in a concordant way and that these methylation differences were consistent with observed differences in gene expression [34]. A methylation signature based on eight genes (*C9orf64, OSMR, MDK, MARVELD1, PTRF, MYD88, BIRC3,* and *RPP25*) was able to predict the survival of GBM patients [35]. Additional abnormally methylated genes have also been identified in GBM: *NPY, TNF, FOXA1, KCNC3,* and *CASP8* [36], and another study identified 251 hypomethylated upregulated genes (Hypo-UGs) and 199 hypermethylated downregulated genes (Hyper-DGs) in GBM [33]. Hypo-UGs were involved in regulating signaling associated with immune responses and infection, while Hyper-DGs were involved in regulating synaptic transmission. Three hub genes for Hyper-DGs were identified: somatostatin, neuropeptide Y, and adenylate cyclase 2. Five hub genes for Hypo-UGs were identified: interleukin-8, matrix metalloproteinase (MMP)-9, cyclin-dependent kinase 1, 2′-5′-oligoadenylate synthetase 1, C-X-C motif chemokine ligand 10, and MMP2. Overexpression of the following Hypo-UGs was significantly associated with poor prognosis in patients: C-type lectin domain containing 5A, epithelial membrane protein 3, solute carrier family 43 member 3, STEAP3 metalloreductase, tumor necrosis factor *α*-induced protein 6, and apolipoprotein B mRNA-editing enzyme catalytic subunit 3G. In contrast to those previous studies, our results suggest that differential expression of the six KDETFs in GBM may not involve differential methylation. These results should be verified in larger datasets and explored further.

The present study may provide new insights into GBM by identifying potential KDETFs and exploring whether differential methylation drives their abnormal expression. At the same time, our conclusions should be treated with caution because our analyses involved a relatively small number of normal samples. In addition, we conducted only bioinformatics analyses, so our results must be verified in experiments with cell lines and clinical samples.

## 5. Conclusions

The present study comprehensively analyzed GBM gene data using bioinformatic methods, which led to the identification of several key differentially expressed TFs, including HOXA3, EN1, ZIC1, FOXD3, HLF, and EGR3. The findings in this study can improve our understanding of the molecular mechanisms underlying GBM and provide potential biomarkers and even therapeutic targets against the disease.

## Data Availability

The data used to support this study are available publicly in The Cancer Genome Atlas (TCGA) database and Gene Expression Omnibus (GEO).

## Supplementary Materials

Supplementary Materials Supplementary Table 1: top 100 up- and downregulated genes. Supplementary Table 2: enrichment of transcription factors based on analysis of differentially expressed genes.
